# Efficacy and safety of mirikizumab in the treatment of inflammatory bowel disease: A meta-analysis

**DOI:** 10.1097/MD.0000000000042123

**Published:** 2025-04-25

**Authors:** Xuemei Chen, Guifei Si, Yuquan Li, Xuemin Yuan

**Affiliations:** a School of Clinical Medicine, Weifang Medical University, Weifang, Shandong, China; b Gastroenterology Department, Linyi People’s Hospital, Linyi, Shandong, China.

**Keywords:** Crohn disease, inflammatory bowel disease, meta-analysis, mirikizumab, ulcerative colitis

## Abstract

**Background:**

This meta-analysis explores the efficacy and safety of mirikizumab in treating IBD.

**Methods:**

A comprehensive search was conducted encompassing randomized controlled trials examining the efficacy of mirikizumab in treating IBD across PubMed, Embase, Cochrane Library, and Web of Science, with a search deadline of November 1, 2023. Quality assessment leaned on the Cochrane manual risk-of-bias evaluation, while Stata 15 undertook the data analysis.

**Results:**

Three randomized controlled studies involving 1602 individuals were finally included. Our meta-analysis suggested that mirikizumab can improve clinical remission (RR = 2.11, 95% CI [1.74, 2.55]), clinical response (RR = 1.68, 95% CI [1.50, 1.89]), endoscopic remission (RR = 1.95, 95% CI [1.65, 2.31]), histologic–endoscopic mucosal improvement (RR = 1.92, 95% CI [1.60, 2.32]) in inflammatory bowel disease (IBD).

**Conclusion:**

According to our meta-analysis, mirikizumab is a promising drug in the treatment of IBD.

## 1. Introduction

Inflammatory bowel disease (IBD) is a chronic, idiopathic inflammatory condition that can affect all parts of the digestive tract, characterized by mucosal immune dysregulation and recurrent bouts of intestinal inflammation.^[1,2]^ Ulcerative colitis (UC) and Crohn disease (CD) represent the 2 expression types of this condition, distinguished by the location and depth of inflammation, closely associated with genetic, immune, lifestyle, and environmental factors.^[3–5]^ Epidemiological studies reveal the highest prevalence of IBD in Europe and North America, with a rapid rise in incidence in emerging industrialized nations.^[6]^ Individuals and their descendants migrating from regions with low IBD prevalence (such as the Middle East and South Asia) to areas with high prevalence exhibit increased susceptibility to IBD. However, the precise causes and mechanisms underlying IBD remain unclear. Inflammation and oxidative stress are generally perceived as key mechanisms in IBD pathogenesis.^[7,8]^ Clinically, treatments for IBD mainly include drugs like 5-aminosalicylic acid, corticosteroids, immunosuppressants (such as azathioprine), and biologics (like anti-TNF-α agents, anti-integrins, and anti-cytokine antibodies).^[9]^ Unfortunately, these medications merely offer symptomatic relief without curing the disease and often lead to noticeable adverse effects such as anemia, liver and kidney dysfunction, leukopenia, cataracts, osteoporosis, malignancies, immunosuppression, and an increased risk of opportunistic infections.^[10]^ Some of these adverse effects are irreversible. Moreover, research indicates that early surgery and the use of immunosuppressants fail to prevent the tendency for reoperation and disease disability in Crohn disease patients.^[11]^ Hence, there is an urgent need to discover safe and effective therapies for IBD.

Developing biologics and small molecule treatments has emerged as a future trend in IBD therapy, showing considerable potential.^[12]^ Genetic studies highlight IL-23 as a key cytokine involved in the mechanisms of UC and CD.^[13]^ Among IL-23 biologics, trials for ustekinumab, risankizumab, and brazikumab in treating CD induction have been completed, and several drugs are under development including guselkumab, tildrakizumab, mirikizumab, and PTG-200.^[14]^ Mirikizumab (developmental code: LY3074828) appears as a promising potential effective treatment, identified as the first anti-IL-23 p19 inhibitor for treating UC and CD patients, including those with acute intestinal symptoms.^[15]^ Mirikizumab, an anti-IL-23 p19 monoclonal antibody, operates by targeting the IL-23 pathway, a pivotal component in the immune response regulation. By blocking the action of the IL-23 p19 subunit, mirikizumab inhibits IL-23 signaling, crucial in regulating the activation and function of specific immune cells like Th17 cells, which play a critical role in the development of inflammatory diseases. Its intervention in the IL-23 signaling pathway modulates the production of inflammatory mediators, potentially reducing inflammation in the gut, offering a promising avenue in treating IBDs by targeting a key immune regulatory pathway implicated in the disease progression.^[16,17]^ However, controversies persist regarding the use of mirikizumab in treating IBD. Therefore, this study aims to address these disputes, offering new choices for clinical management.

## 2. Method

The systematic review described herein was accepted by the online PROSPERO international prospective register of systematic reviews^[18]^ of the National Institute for Health Research (CRD42023483745).

### 2.1. Inclusion and exclusion criteria

The included population met the diagnostic criteria for IBD.^[19]^ Mirikizumab was used in the experimental group and placebo was used in the control group, and the primary outcome were clinical remission; clinical response; endoscopic remission; and the secondary outcome were histologic–endoscopic mucosal improvement; adverse events, the randomized controlled trial was included in this study.

Conference abstracts, meta-analyses, systematic reviews, animal experiments, Full text is not available and case reports will be considered for exclusion.

### 2.2. Literature retrieval

Randomized controlled trials on mirikizumab for IBD were searched in PubMed, Embase, Cochrane Library, Web of science, with a search deadline of November 1, 2023, using the mesh word combined with a free word: Mirikizumab IBD. Detailed search strategies are provided in Table S1, Supplemental Digital Content, https://links.lww.com/MD/O707.

### 2.3. Data extract

Two authors rigorously screened the literature based on predetermined inclusion and exclusion criteria. In case of any disagreement, they resolved it through discussion or sought the opinion of a third party to negotiate and reach consensus. Information extracted from the included studies included the following key details: authors, year, NCT number, country, sample size, gender, mean age, Type of disease, intervention, and outcome.

### 2.4. Grade of evidence

To determine the quality of our results, we selected the Graded Recommendations Assessment Development and Evaluation system to evaluate the evidence^[20]^ for methodological quality. We considered 5 factors that could reduce the quality of the evidence, including study limitations, inconsistent findings, inconclusive direct evidence, inaccurate or wide CIs, and publication bias. In addition, 3 factors that could reduce the quality of evidence were reviewed, namely effect size, possible confounding factors, and dose-effect relationships. A comprehensive description of the quality of evidence for each parameter data is provided (Table S2, Supplemental Digital Content, https://links.lww.com/MD/O708).

### 2.5. Included studies’ risk of bias

Two investigators independently assessed the risk of bias as low, unclear, or high using the Cochrane Collaboration tools.^[21]^ If there was any disagreement, a third person was consulted to reach consensus. The assessment included 7 areas: generation of randomized sequences (selective bias), allocation concealment (selective bias), blinding of implementers and participants (implementation bias), blinding of outcome assessors (observational bias), completeness of outcome data (follow-up bias), selective reporting of study results (reporting bias), and other potential sources of bias. Each included study was assessed individually against these criteria. If a study fully met all criteria, it was at “low risk” of bias, indicating a high-quality study and low overall risk of bias. If a study partially met the criteria, its quality was categorized as “unclear risk,” indicating a moderate likelihood of bias. If a study did not meet the criteria at all, it was categorized as “high risk,” indicating a high risk of bias and low quality of the study.

### 2.6. Data analysis

The collected data were statistically analyzed using Stata 15.0 software (Stata Corp, College Station). Heterogeneity between included studies was assessed using *I*^2^ values or Q-statistics. *I*^2^ values of 0%, 25%, 50%, and 75% indicated no heterogeneity, low heterogeneity, moderate heterogeneity, and high heterogeneity, respectively. If the *I*^2^ value was equal to or >50%, a sensitivity analysis was performed to explore potential sources of heterogeneity. If heterogeneity was <50%, analyses were conducted using a fixed-effects model. Standardized mean difference and 95% confidence interval (CI) were used for continuous variables and risk ratio (RR) and 95% CI for dichotomous variables. In addition, random effects model and Egger test were used to assess publication bias.

## 3. Result

### 3.1. Study selection

**Figure 1.**
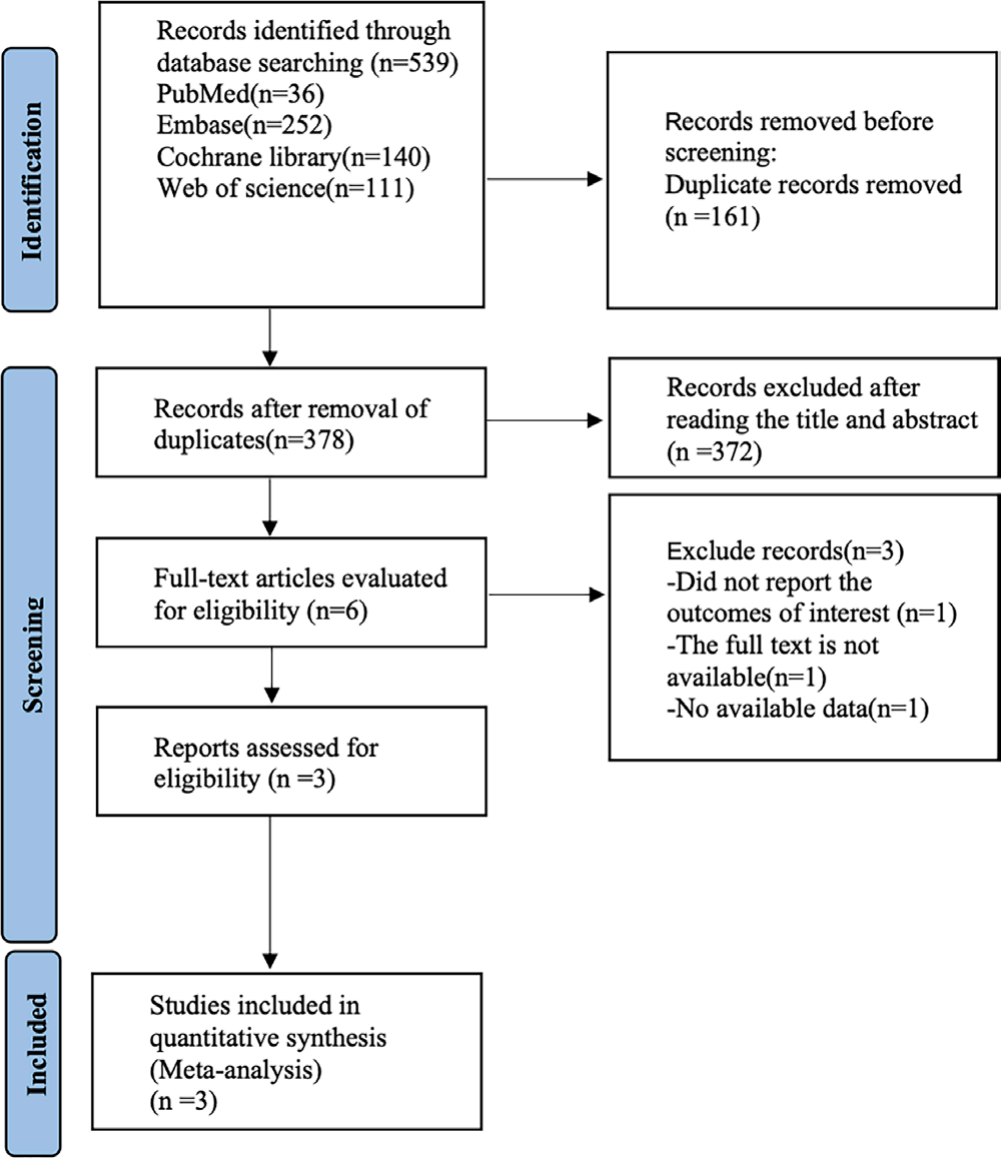
Literature search flow chart.

Figure 1 shows our literature search process, which initially retrieved 539 documents, removed 161 duplicates, removed 372 articles by reading titles and abstracts, removed 3 papers by reading the full text, and finally included 3 randomized controlled trials^[22–24]^ for analysis.

### 3.2. Basic characteristics and risk of bias of the included studies

Three randomized controlled studies involving 1602 individuals were finally included, in 2 articles^[22,23]^ for UC and one^[24]^ for Crohn disease, doses of mirikizumab ranged from 50 to 1000 mg. Baseline characteristics are shown in Table 1 The 4 included studies clearly accounted for the method of randomization used, and the risk of bias results are shown in Figure S1–S2, Supplemental Digital Content, https://links.lww.com/MD/O709.

**Table 1:** Baseline characteristics table.

Study	Year	NCT	Country	Sample size	Gender (M/F)	Mean age (yr)	Type of disease	Intervention	Outcome
D’Haens^[23]^	2023	NCT03518086 NCT03524092	USA	Mirikizumab: 868 Placebo: 294	695/467	Mirikizumab: 42.9 Placebo: 41.3	Ulcerative Colitis	Mirikizumab: 300 mg intravenously, every 4 wk	F1; F2; F3; F4; F5
Sands^[24]^	2022	NCT02891226	USA	Mirikizumab 200 mg: 31 Mirikizumab 600 mg: 32 Mirikizumab 1000 mg: 64 Placebo: 64	93/98	Mirikizumab 200 mg: 38.1 Mirikizumab 600 mg: 40 Mirikizumab 1000 mg: 37.7 Placebo: 39	Crohn Disease	Mirikizumab: 200; 600; 1000 mg intravenously, every 4 wk	F1; F2; F3; F5
Sandborn^[22]^	2020	NCT02589665	USA	Mirikizumab 50 mg: 63 Mirikizumab 200 mg: 62 Mirikizumab 600 mg: 61 Placebo: 63	149/100	Mirikizumab 50 mg: 25 Mirikizumab 200 mg: 25 Mirikizumab 600 mg: 23 Placebo: 27	Ulcerative Colitis	Mirikizumab: 200; 600; 50 mg intravenously, every 4 wk	F1; F2; F3; F4; F5

F1 = clinical remission, F2 = clinical response, F3 = endoscopic remission, F4 = histologic–endoscopic mucosal improvement, F5 = adverse events, M/F = male/female.

### 3.3. Result of meta-analysis

#### 3.3.1. Clinical remission

**Figure 2.**
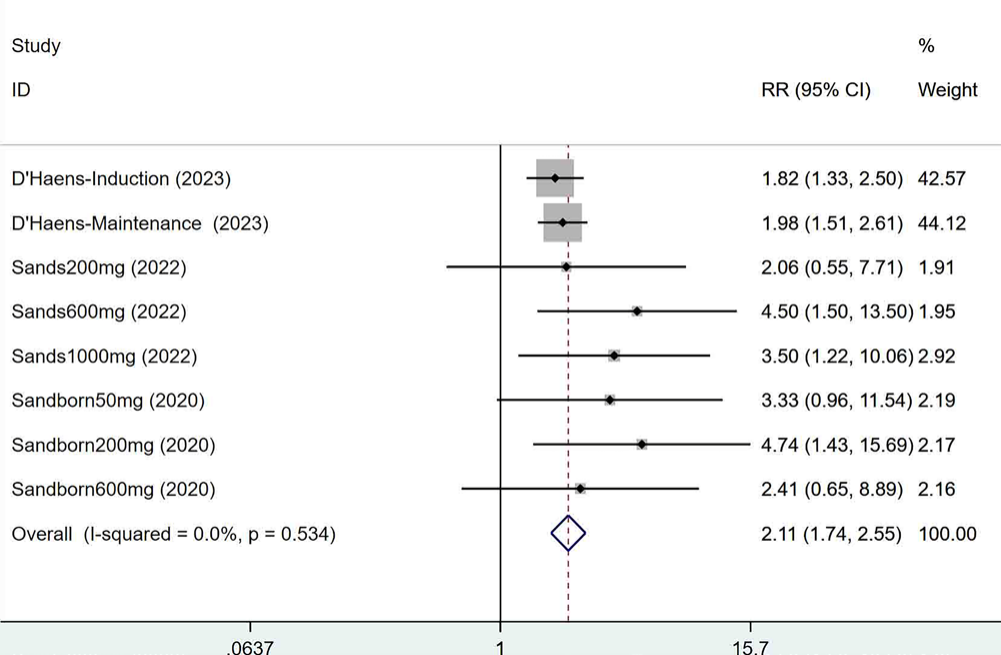
Forest plot of meta-analysis of clinical remission.

Three articles mentioned the clinical remission, which was tested for heterogeneity (*I*^2^ = 0%, *P* = .534), therefore fixed effects model was used. The results of the analysis (Fig. 2) suggested that mirikizumab can improve clinical remission in IBD (RR = 2.11, 95% CI [1.74, 2.55]).

#### 3.3.2. Clinical response

**Figure 3.**
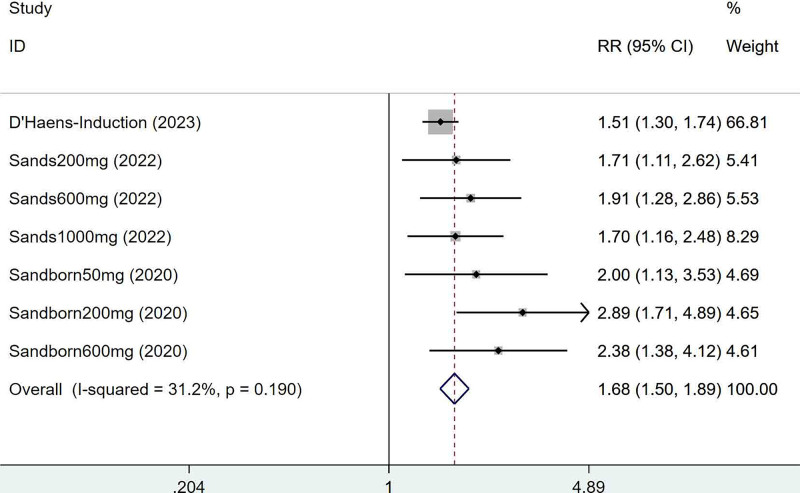
Forest plot of meta-analysis of clinical response.

Three articles mentioned the clinical response, which was tested for heterogeneity (*I*^2^ = 31.2%, *P* = .190), therefore fixed effects model was used. The results of the analysis (Fig. 3) suggested that mirikizumab can improve clinical response in IBD (RR = 1.68, 95% CI [1.50, 1.89]).

#### 3.3.3. Endoscopic remission

**Figure 4.**
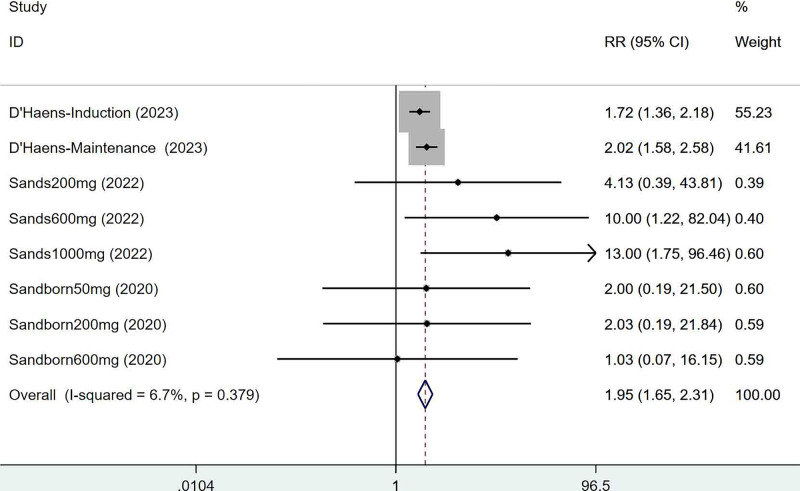
Forest plot of meta-analysis of endoscopic remission.

Three articles mentioned the endoscopic remission, which was tested for heterogeneity (*I*^2^ = 6.7%, *P* = .379), therefore fixed effects model was used. The results of the analysis (Fig. 4) suggested that mirikizumab can improve endoscopic remission in IBD (RR = 1.95, 95% CI [1.65, 2.31]).

#### 3.3.4. Histologic–endoscopic mucosal improvement

**Figure 5.**
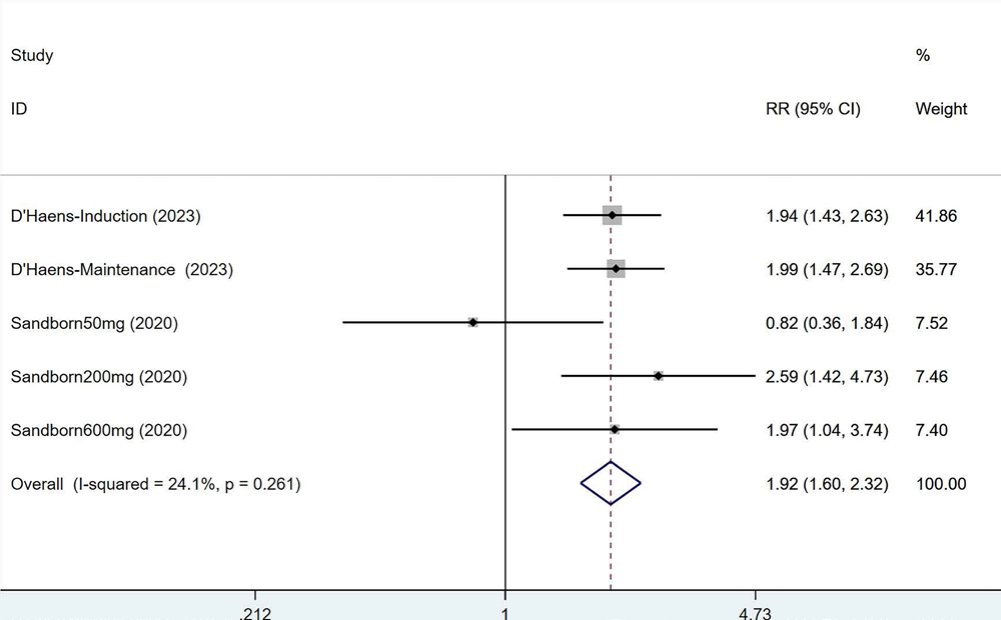
Forest plot of meta-analysis of histologic–endoscopic mucosal improvement.

Two articles mentioned the histologic–endoscopic mucosal improvement, which was tested for heterogeneity (*I*^2^ = 24.1%, *P* = .261), therefore fixed effects model was used. The results of the analysis (Fig. 5) suggested that mirikizumab can improve histologic–endoscopic mucosal improvement in IBD (RR = 1.92, 95% CI [1.60, 2.32]).

#### 3.3.5. Adverse events

Three articles mentioned the adverse events, including nasopharyngitis, arthraigia, worsening, headache, anemia. Results (Table 2) suggest that mirikizumab can prevent the deterioration of IBD (RR = 0.27, 95% CI [0.19, 0.37]), but there is no significant difference in other adverse effects.

**Table 2:** Meta-analysis of adverse events.

Adverse events	No of study	Heterogeneity		RR (95% CI)	*P*
		*I*^2^ (%)	*P*		
Nasopharyngitis	8	0	.703	1.17 (0.8, 1.69)	.420
Arthraigia	5	0	.874	1.39 (0.81,2.39)	.231
Worsening	8	0	.907	0.27 (0.19,0.37)	.0001
Headache	8	11	.345	1’07 (0.70,0.63)	.758
Anemia	8	0	.571	0.68 (1.46,1.02)	.60

CI = confidence interval, RR = risk ratio.

#### 3.3.6. Published bias

Publication bias was assessed by an Egger’s test for clinical remission, clinical response, endoscopic remission, histologic–endoscopic mucosal improvement, adverse events. Which showed no publication bias for change endoscopic remission in Supplementary Material Figure S3 (*P* = .150), Figure S3, Supplemental Digital Content , https://links.lww.com/MD/O709, histologic–endoscopic mucosal improvement in Supplementary Material Figure S4 (*P* = .500), Figure S4, Supplemental Digital Content, https://links.lww.com/MD/O709, adverse events in Supplementary Material Figure S5 (*P* = .133), Figure S5, Supplemental Digital Content, https://links.lww.com/MD/O709, However, publication bias was detected in clinical remission in Supplementary Material Figure S6 (*P* = .015), Figure S6, Supplemental Digital Content, https://links.lww.com/MD/O709 and clinical response in Supplementary Material Figure S7 (*P* = .011), Figure S7, Supplemental Digital Content, https://links.lww.com/MD/O709.

## 4. Discussion

As far as we know, this marks the inaugural use of meta-analysis to assess the efficacy and safety of mirikizumab for IBD. Our study reveals that mirikizumab has the capability to enhance clinical remission, clinical response, endoscopic remission, and histologic–endoscopic mucosal improvement indicators in IBD patients. Research indicates a significant elevation in IL-23p19-positive cells and IL-23 mRNA levels within intestinal mucosal tissues among individuals with IBD.^[25,26]^ IL-23 notably triggers lymphocyte responses in IBD patients and activates peripheral blood mononuclear cells, elevating the secretion levels of inflammatory factors like TNF-α, IFN-γ, and IL-2, thus prompting inflammatory reactions in the intestinal mucosa – a primary mechanism in autoimmune inflammatory diseases and chronic enteritis.^[27,28]^ However, the binding of mirikizumab to the p19 subunit of IL-23 obstructs the IL-23/Th17 pathway, reducing the concentration of IL-23 and the expression of Th17 and its associated cytokines.^[29]^ This reduction diminishes cytokine-mediated intestinal inflammation without affecting systemic T-cell responses. Consequently, it manages and alleviates the progression of intestinal inflammation, achieving symptomatic control (clinical remission) and suppressing intestinal inflammation (endoscopic remission).^[30,31]^ Yet, current research on mirikizumab relationship with IBD is limited, with numerous ongoing NCTs. Hence, we eagerly anticipate further studies to substantiate our conclusions.

Moreover, our research findings indicate that mirikizumab does not escalate adverse reactions, aligning with results from an earlier Phase I trial. In this study,^[32]^ 60 subjects aged 18 to 65 were randomly allocated into observation and control groups. The observation group received 5 treatment regimens: three via intravenous mirikizumab administration (300, 600, and 1200 mg) and 2 via subcutaneous injection (200 and 400 mg). Meanwhile, the control group received corresponding doses of buffer solution (placebo) devoid of active ingredients – three via intravenous administration (300, 600, and 1200 mg) and 2 via subcutaneous injection (200 and 400 mg). Over a span from day 0 to day 85 post-administration, the incidence rate of adverse reactions and pain scores on the Visual Analogue Scale, the 2 primary endpoints, remained within the bounds of safety. This conclusion further bolsters the credibility of our study.

Our study still has several limitations: firstly, there is some heterogeneity due to the small number of included studies and the inclusion of studies including Crohn and UC. Secondly, due to the limitation of the number of included studies, we were not able to perform subgroup analyses for outcomes with large heterogeneity. Finally, the dose and duration of time used for mirikizumab were also inconsistent, which may also contribute to the source of heterogeneity.

## 5. Conclusion

Mirikizumab is a promising drug in the treatment of IBD, but due to the limitations of our study, more high-quality, multicenter, large sample, randomized controlled studies are necessary to further support our findings.

## Author contributions

**Conceptualization:** Xuemei Chen, Guifei Si, Xuemin Yuan.

**Data curation:** Xuemei Chen, Guifei Si, Yuquan Li, Xuemin Yuan.

**Formal analysis:** Xuemei Chen, Guifei Si, Yuquan Li, Xuemin Yuan.

**Funding acquisition:** Guifei Si, Yuquan Li, Xuemin Yuan.

**Investigation:** Xuemei Chen, Guifei Si, Yuquan Li, Xuemin Yuan.

**Methodology:** Yuquan Li, Xuemin Yuan.

**Project administration:** Xuemei Chen, Guifei Si, Xuemin Yuan.

**Resources:** Xuemei Chen, Xuemin Yuan.

**Software:** Yuquan Li, Xuemin Yuan.

**Supervision:** Yuquan Li, Xuemin Yuan.

**Validation:** Guifei Si, Yuquan Li, Xuemin Yuan.

**Visualization:** Guifei Si, Xuemin Yuan.

**Writing – original draft:** Guifei Si, Xuemin Yuan.

## Supplementary Material


