# Supplementary material for: Efficacy and safety of mirikizumab in the treatment of inflammatory bowel disease: A meta-analysis
Source: Medicine (Baltimore). 2025 Apr 25;104(17):e42123. doi: 10.1097/MD.0000000000042123 (PMC12039982; doi:10.1097/MD.0000000000042123)
Supplement: Supplementary file 1 [file medi-104-e42123-s001.docx]

PubMed

(("mirikizumab" [Supplementary Concept]) OR (((Mirikizumab[Title/Abstract]) OR (LY-3074828[Title/Abstract])) OR (LY3074828[Title/Abstract]))) AND (("Inflammatory Bowel Diseases"[Mesh]) OR (((((Inflammatory Bowel Disease[Title/Abstract]) OR (Inflammatory Bowel Diseases[Title/Abstract])) OR (Bowel Diseases, Inflammatory[Title/Abstract])) OR (Ulcerative Colitis[Title/Abstract])) OR (Crohn Disease[Title/Abstract])))
