# Supplementary material for: Efficacy and safety of mirikizumab in the treatment of inflammatory bowel disease: A meta-analysis
Source: Medicine (Baltimore). 2025 Apr 25;104(17):e42123. doi: 10.1097/MD.0000000000042123 (PMC12039982; doi:10.1097/MD.0000000000042123)
Supplement: Supplementary file 2 [file medi-104-e42123-s002.docx]

Table S1 Grade of evidence

| Outcome | Grade |
| --- | --- |
| Clinical Remission | Moderate |
| Clinical Response | Moderate |
| Endoscopic Remission | Low |
| Histologic–Endoscopic Mucosal Improvement | Low |
| adverse events | Low |
