# Supplementary material for: Efficacy and safety of mirikizumab in the treatment of inflammatory bowel disease: A meta-analysis
Source: Medicine (Baltimore). 2025 Apr 25;104(17):e42123. doi: 10.1097/MD.0000000000042123 (PMC12039982; doi:10.1097/MD.0000000000042123)

Figure s1 risk bias of graph


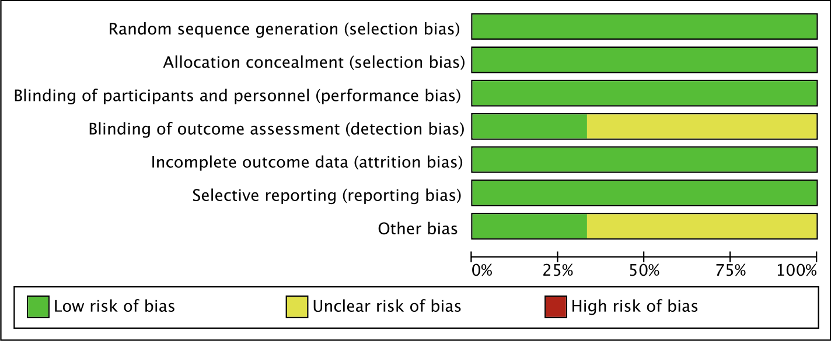


Figure s2 risk bias of summary


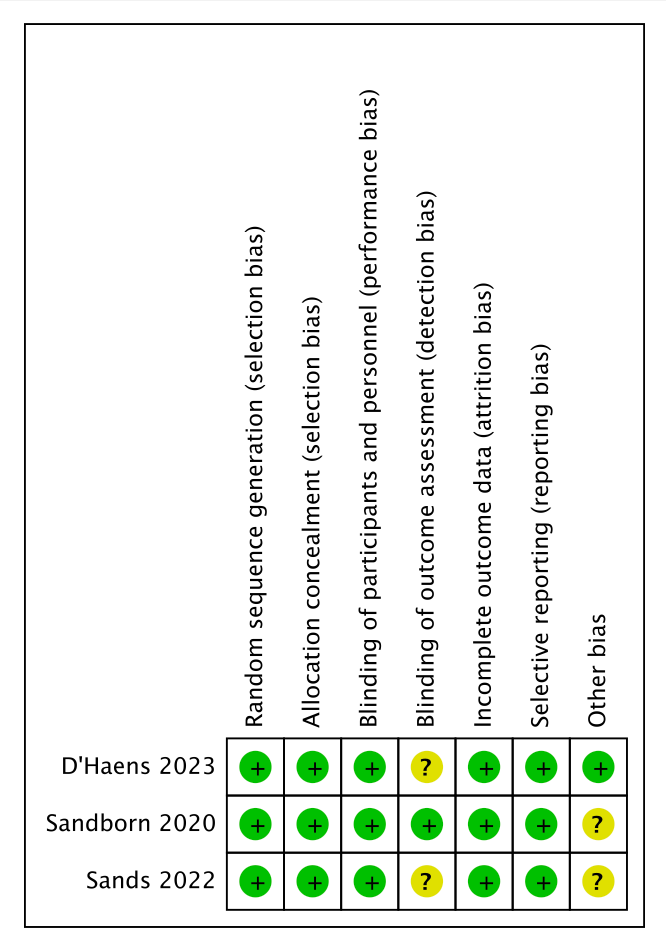


Figure S3 egger test of endoscopic remission


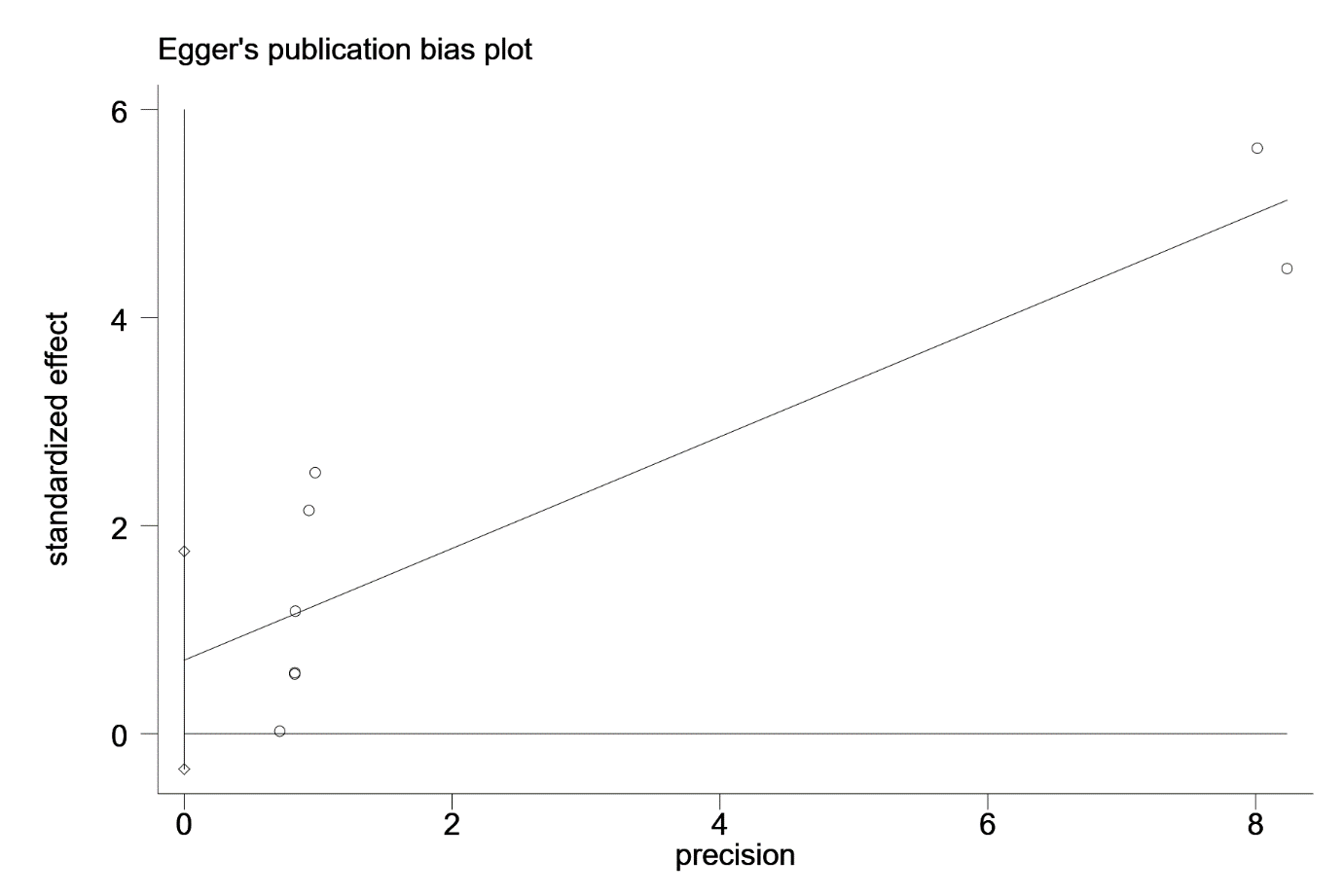


Figure S4 egger test of histologic–endoscopic mucosal improvement


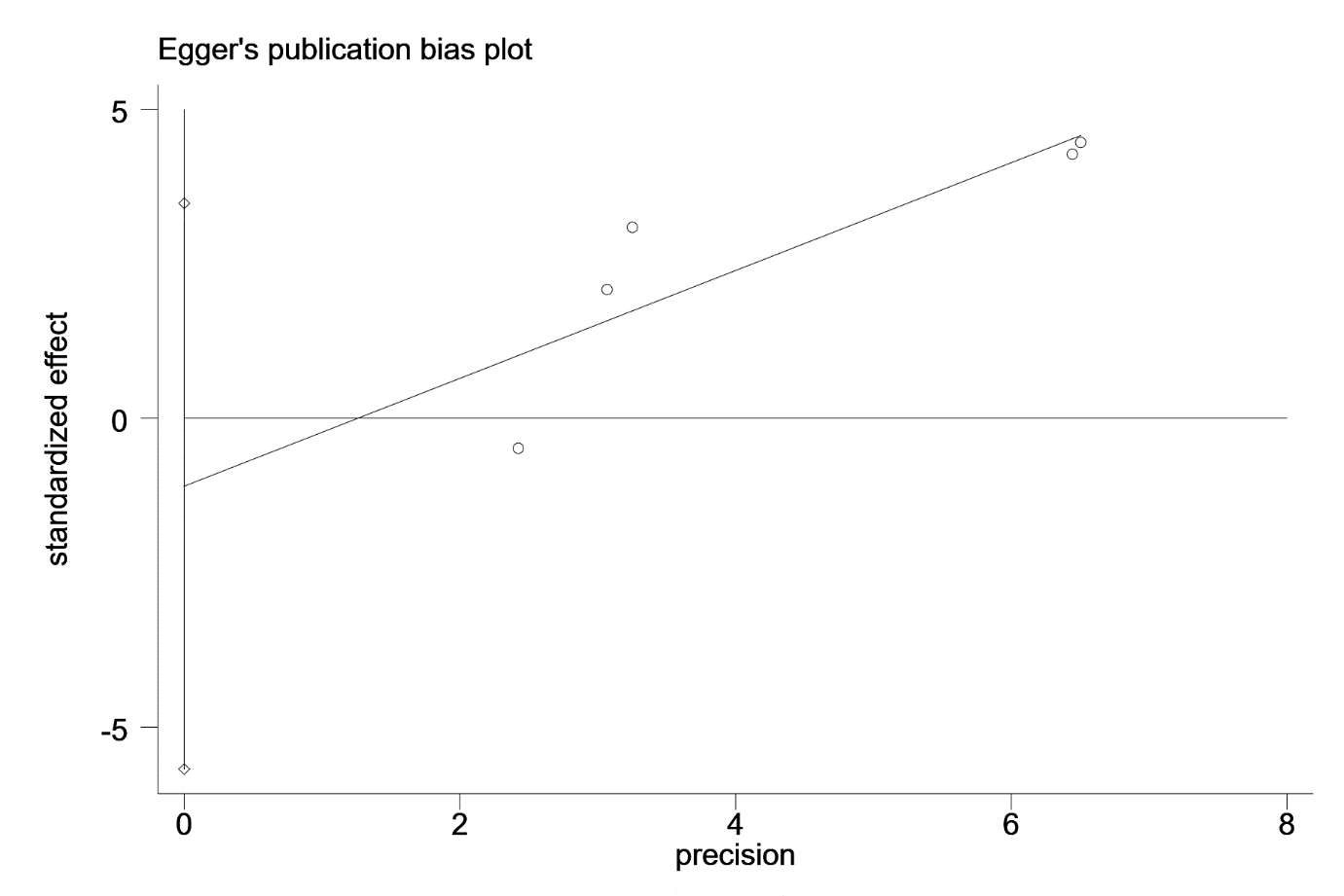


Figure S5 egger test of adverse events


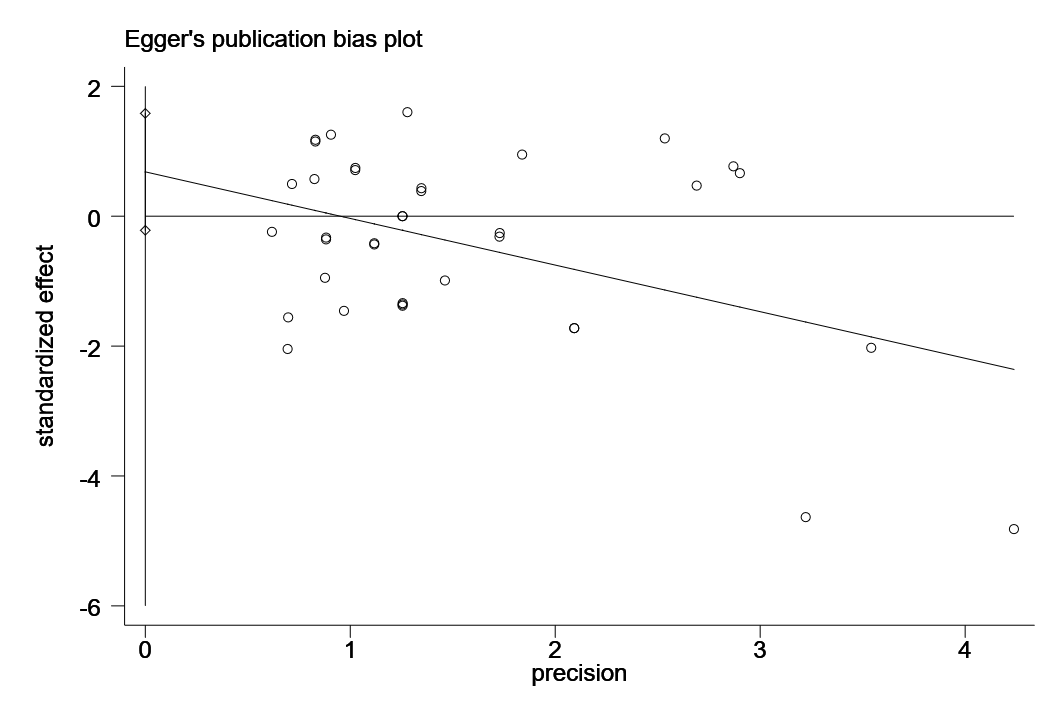


Figure S6 egger test of clinical remission


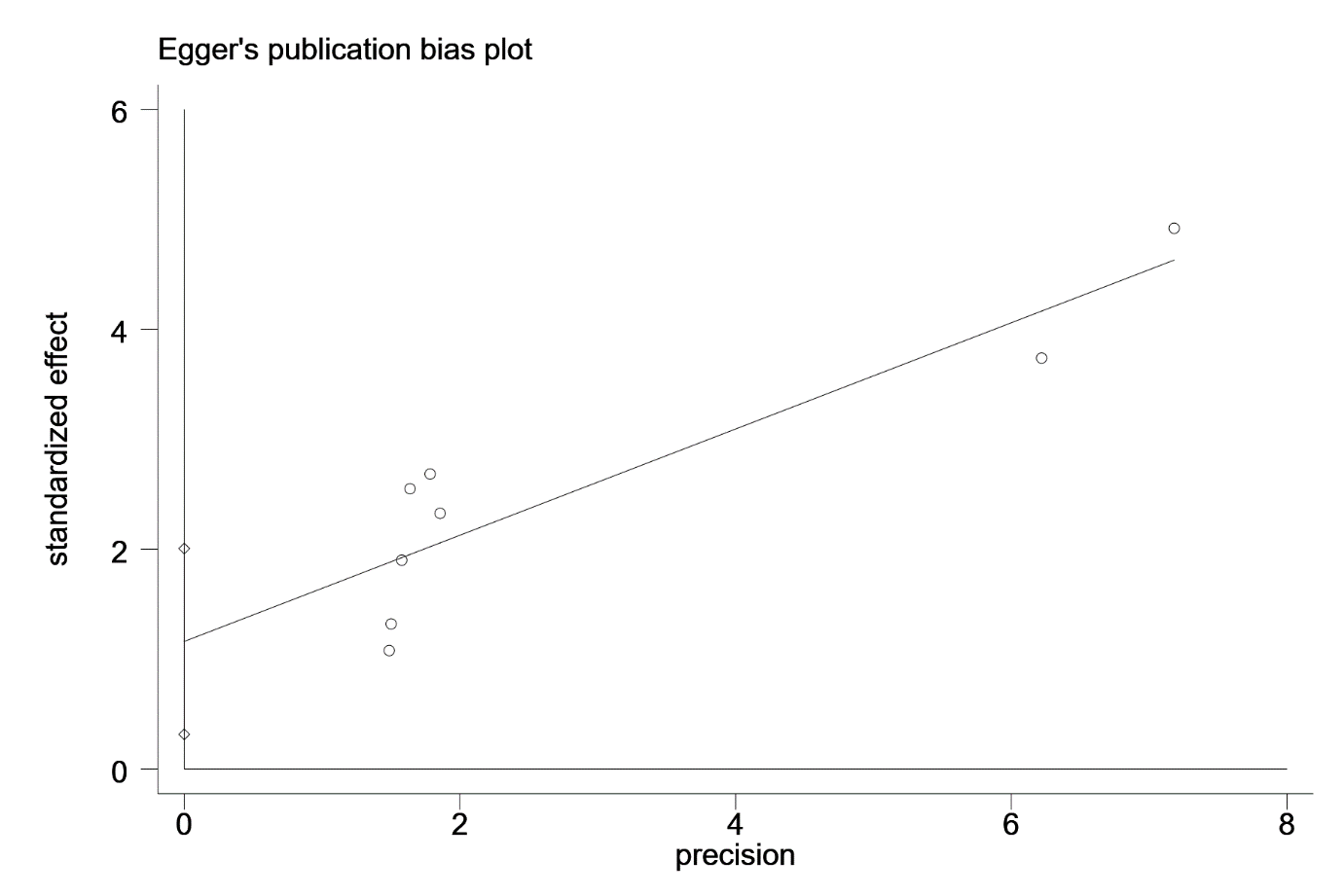


Figure S7 egger test of clinical response


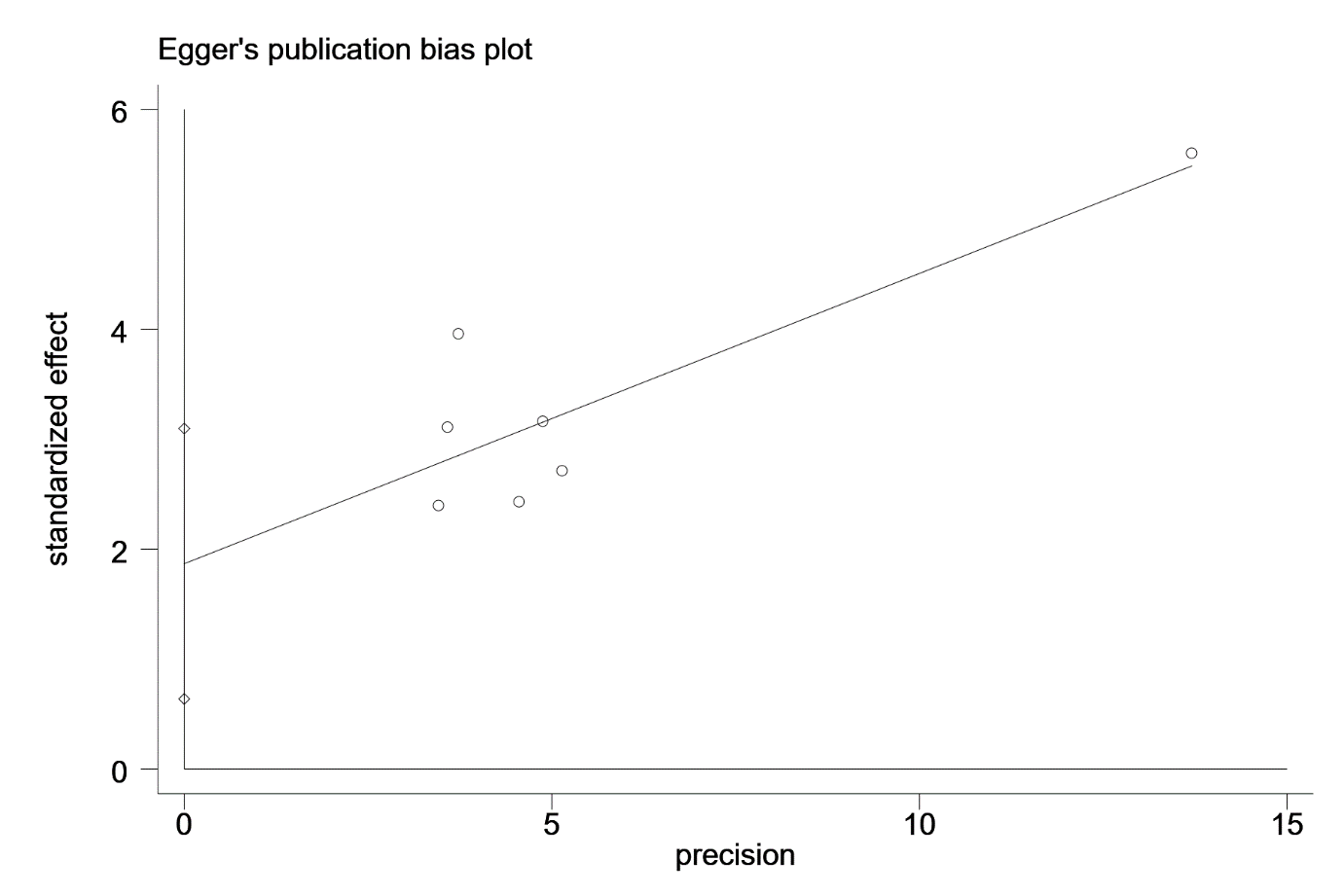

Supplement: Supplementary file 3 [file medi-104-e42123-s003.docx]
